# The association between flooring materials and childhood asthma: A prospective birth cohort in the Japan Environment and Children’s Study

**DOI:** 10.1371/journal.pone.0305957

**Published:** 2024-07-31

**Authors:** Hiroyoshi Iwata, Atsuko Ikeda, Mariko Itoh, Sachiko Itoh, Rahel Mesfin Ketema, Naomi Tamura, Chihiro Miyashita, Takeshi Yamaguchi, Keiko Yamazaki, Rieko Yamamoto, Maki Tojo, Yasuaki Saijo, Yoshiya Ito, Reiko Kishi, the Japan Environment and Children’s Study (JECS) Group

**Affiliations:** 1 Center for Environmental and Health Sciences, Hokkaido University, Sapporo, Japan; 2 Faculty of Health Sciences, Hokkaido University, Sapporo, Japan; 3 Division of Public Health and Epidemiology, Department of Social Medicine, Asahikawa Medical University, Asahikawa, Japan; 4 Faculty of Nursing, Japanese Red Cross Hokkaido College of Nursing, Kitami, Japan; Satyawati College, University of Delhi, INDIA

## Abstract

**Background:**

Childhood asthma is known to be affected by a range of factors, including conditions in the indoor environment. While flooring material influences indoor air conditions, the potential association between flooring materials and childhood asthma remains poorly understood in Japan.

**Objective:**

The present study aims to assess the association between childhood asthma incidence and the primary flooring material with the ongoing prospective nationwide birth cohort data of the Japan Environment and Children’s Study (JECS).

**Methods:**

The JECS gathered data on mothers and children through 15 Regional Centres across Japan. The present study assessed flooring materials used in the home and asthma incidence at age four among children born between 2011 and 2014. We implemented logistic regressions, setting asthma incidence among the children as the outcome and home floor type as the exposure. Additional analyses were conducted, stratifying the home’s age as a proxy for tatami age, to assess whether the potential effect of tatami flooring on asthma risk is influenced by its age.

**Results:**

The present study included total of 75,629 infants. For tatami flooring, the main multivariable regression and additional sub-group regression for homes over ten years old produced odds ratios of 1.09; 95% Confidence Interval (CI) [1.01–1.17] and 1.10; 95% CI [1.00–1.21] compared with flooring, respectively.

**Conclusion:**

These results imply that exposure to tatami flooring, particularly in older homes, may be associated with childhood asthma incidence. Moreover, our study highlights the importance of evaluating the relationship between regional and cultural differences between asthma and flooring materials.

## 1. Introduction

Childhood asthma affects a tremendous number of patients across the world. Over the past 40 years, rates of asthma among children increased [1]. The World Health Organization (WHO) estimated in the early 2000’s that about 300 million patients had asthma worldwide, with this number predicted to climb to 400 million by 2025 [2]. Asthma caused over 450,000 deaths worldwide in 2019 [3]. Furthermore, asthmatic children tend to have more difficulty than adults when using an inhaler, underscoring the importance of preventing childhood asthma [3].

A variety of environmental risk factors for asthma have been identified, including second hand smoke, household pets, and mold [4]. The indoor environment is of particular importance due to the large amounts of time typically spent indoors. Accordingly, many studies have investigated the indoor risk factors for asthma. In particular, flooring materials were reviewed by Becher et al. [5], who reported increased risks of asthma associated with carpeting compared to hard flooring in Sweden and Denmark [6, 7]. More recently, dampness has been reported as an asthma risk factor [8, 9].

Importantly, trends in flooring materials vary greatly from country to country. Traditional Japanese customs around flooring have two key characteristics: first, the custom of removing shoes when entering living spaces is nearly universal; second, many homes are equipped with traditional flooring mats called tatami. Tatami is mainly made of Japanese straw "*Igusa*," having a thickness of around 50 mm and cushioning properties. The elasticity and cushioning properties of tatami have been cited as reasons why it is favored as a means of preventing fall injuries among infants and children [10]. Even in the 21^st^ century, a housing company survey found that about 70% of new houses in Japan had at least one room with tatami flooring [11]. Further, many Japanese homes use carpet over tatami, suggesting that there are four main flooring styles in Japan: hard flooring (wood, vinyl, tile, etc.), carpet, tatami, and carpet over tatami. Hence, overseas findings suggesting that carpet poses a risk of asthma when compared to hard flooring may not be directly applicable to Japanese living spaces.

Although the evidence is inconclusive, some studies have suggested that tatami flooring merits investigation as a potential risk factor for childhood asthma. Hamada et al. (2007) reported that higher levels of fungi were detected in tatami dust, which contains more moisture, in comparison with carpet dust [12]. Sugiyama et al. (2002) reported that, among 13-to-14-year-old Japanese children complaining of severe wheezing, more lived with tatami flooring than carpeted flooring [13]. Nishima et al. later reported that tatami flooring increased the prevalence of wheezing in a study of about 37,000 elementary school children [14]. Importantly, both Sugiyama et al. and Nishima et al. assessed wheezing rather than the incidence of childhood asthma. Although most asthmatic children do experience wheezing during exacerbations, childhood wheezing is a nonspecific finding which may be present in a range of diagnoses other than asthma [15]. As of this writing, the authors are unaware of any studies that have directly assessed the potential association between common Japanese flooring materials, including tatami, and the incidence of childhood asthma.

The present study aims to investigate the association between childhood asthma incidence and the four types of flooring most common in Japan–hard flooring, carpet over hard flooring, tatami, and carpet over tatami–using data from children gathered in the Japan Environment and Children’s Study (JECS), an ongoing nationwide birth cohort study with over 100,000 participants. While the present study is specifically focused on Japanese flooring materials, our findings may illustrate the importance of location-specific investigations of the risk profiles of various flooring materials in relation to childhood asthma.

## 2. Methods

### 2.1. Study design and population

**Fig 1:**
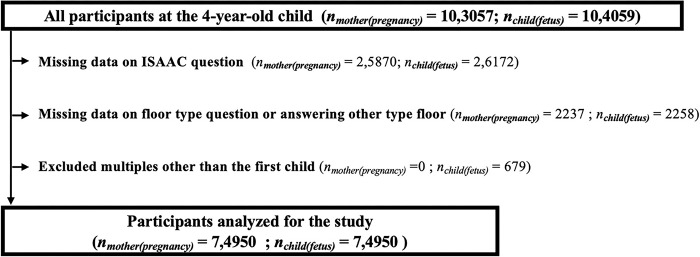
Study participant inclusion flow chart.

The present study used data from the JECS, an ongoing prospective birth cohort study. The precise methodology of the JECS is presented in Kawamoto et al. (Kawamoto et al., 2014). In summary, the JECS recruited pregnant mothers from 15 national Regional Centres across Japan from January 2011 to March 2014, including over 100,000 pregnancies (jecs-ta-20190930 and jecs-qa-20210401 datasets). The study flow chart is shown in **Fig 1**, suggesting we analyzed 74950 research participants. Our study included all 4-year-old participants of the JECS study. We excluded those who did not answer the asthma question, or answered floor question inadequately, or were multiples other than the first child.

### 2.2. Assessment of indoor floor materials

We used data from a JECS questionnaire survey completed during the mothers’ second or third-trimester pregnancy, which asked mothers to report mainly the flooring material used in their homes. Respondents selected from the following options: tatami, carpet over tatami, hard flooring, carpet over hard flooring, or other [16]. We excluded respondents who selected “other” due to the high likelihood of heterogeneity within that group.

### 2.3. Childhood asthma incidence follow-up

The primary outcome was the incidence of asthma from infancy to age four. The JECS ascertained the incidence of childhood asthma by asking the caregivers Question 6 from the International Study of Asthma and Allergies in Childhood (ISAAC): "Has your child/have you ever had asthma?" [17–23] Asthma data were collected periodically using self-administered questionnaires completed over the term when the infant was 6 months to 4 years old. We used the responses gathered at age four as a measure of asthma incidence from birth up to that point.

### 2.4. Statistical analysis

We first performed descriptive statistics to assess the basic characteristics of mothers and children, as well as their living conditions. Secondly, we conducted chi-square tests on asthma incidence and floor materials among all participants and within each sub-group. We then implemented univariable and multivariable logistic regressions using incidence of childhood asthma as the outcome variable. The principle independent variables were flooring conditions: hard flooring was set as the reference, and carpet over hard flooring, tatami, and carpet over tatami were the factor variables. We conducted the multivariable regressions adjusting for covariates. The statistical analyses were performed with performed using R software (version 4.0.3; R Foundation for Statistical Computing, Vienna, Austria).

### 2.5. Covariates

**Fig 2:**
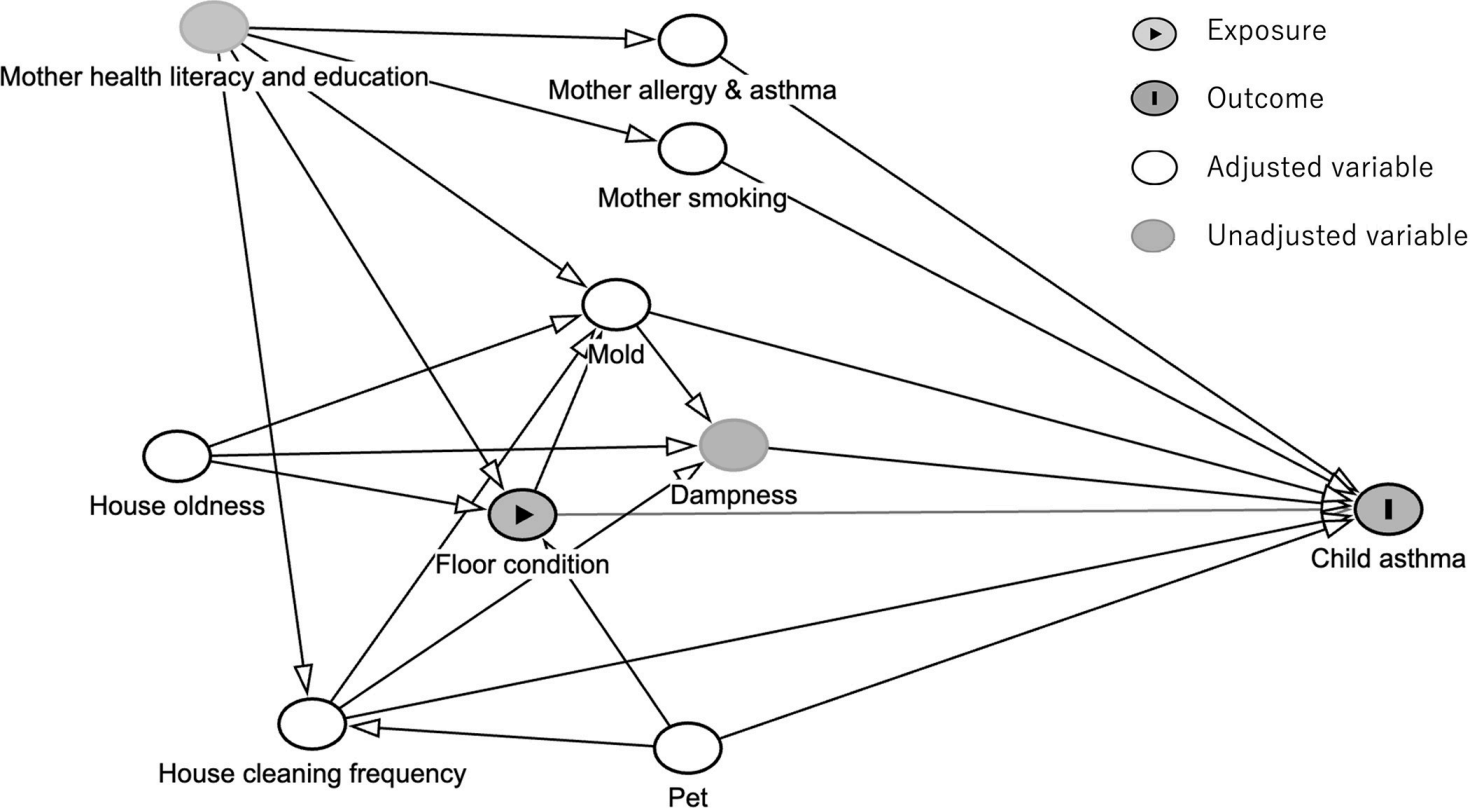
Directed acyclic graph in the present study.

We collected information about potential covariates in light of previous research into childhood asthma incidence. Next, we built a directed acyclic graph (DAG) (**Fig 2**) to help assess which potential confounders to adjust [24, 25]. We identified the following potential covariates: maternal allergy and asthma history, maternal smoking history, frequency of home cleaning, history of pet ownership (including dogs, cats, and birds), and presence of mold on floors. We regarded maternal health literacy and mother education level as the representative of social economic status (SES) [26–28]. However, we did not adjust maternal health literacy and education level directly as a covariate in regressions because the effect were adjusted via covariates: maternal allergy and asthma history, maternal smoking history, frequency of home cleaning, and presence of mold on floors. based on our DAG (**Fig 2**). Data on covariates were also collected using self-administered questionnaires.

### 2.6. Additional analysis

We considered that the age of the tatami flooring may affect the risk of childhood asthma. However, as the JECS questionnaire did not gather information about flooring age, we used the age of the home as a proxy for tatami age, since older homes tend to have older tatami [29]. Participants were stratified into two groups based on home age, using ten years as the cutoff value [30]. Moreover, in 2003, about ten years ago, the research participants’ recruitment, Japan’s revised Building Standard Act was enacted and forced all living rooms to be capable of 24-hour ventilation in awareness of importance of lowering indoor pollutant concentrations. If the research participants answered, "do not know their house age," the house was highly suspected to be old, we sorted them into groups whose houses were over ten years old for the analysis.

### 2.7. Ethics approval and consent to participate

The JECS protocol was reviewed and approved by the Ministry of the Environment’s Institutional Review Board on Epidemiological Studies (No. 100910001), and the Ethics Committees of all participating institutions. The JECS obtained written informed consent from all study participants. Moreover, the JECS followed the Declaration of Helsinki and its revisions. Written informed consent for participation in the study were obtained from individual mothers and their partners, and for children from their parent or guardian [31].

## 3. Results

**Fig 3:**
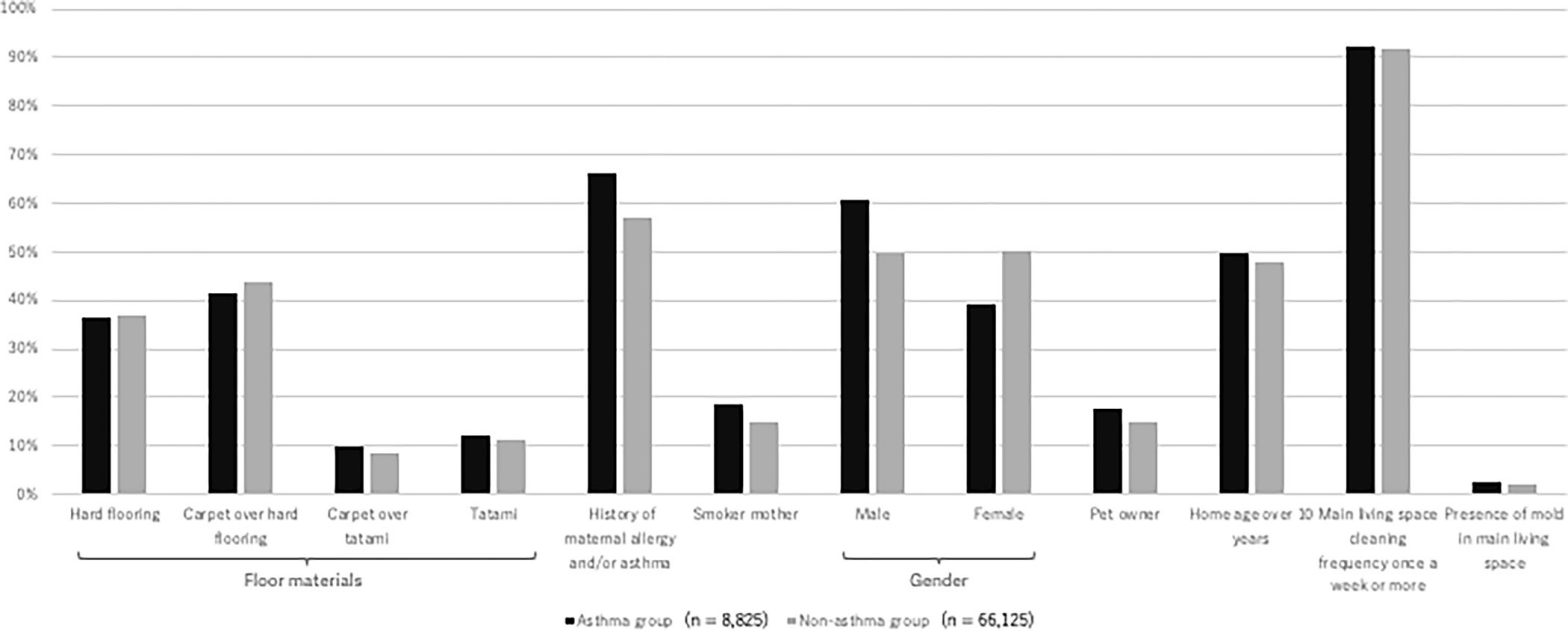
Comparison of asthma and non-asthma group characteristics.

We summarize the basic characteristics of study participants in **Table 1 and Fig 3**. There were no appreciable differences in maternal age between the asthma and non-asthma groups; median, first and third quartile values were 31.0 [28.0, 35.0] years for both groups. Both smoker status and history of maternal allergy and asthma were more frequent in the asthma group than the non-asthma group. Also, the incidence of asthma was higher among male children. The most frequently reported flooring type was hard flooring, followed by carpet over hard flooring, tatami, and carpet over tatami.

**Table 1:** Study participant characteristics.

	Asthma group	Non-asthma group	Overall
	(N = 8825)	(N = 66125)	(N = 74950)
**Flooring type in main living space**			
Hard flooring	3224 (36.5%)	24278 (36.7%)	27502 (36.7%)
Carpet over hard flooring	3672 (41.6%)	28806 (43.6%)	32478 (43.3%)
Carpet over tatami	863 (9.8%)	5681 (8.6%)	6544 (8.7%)
Tatami	1066 (12.1%)	7360 (11.1%)	8426 (11.2%)
**Mother age at birth, years**			
Median [Q1, Q3]	31.0 [28.0, 35.0]	31.0 [28.0, 35.0]	31.0 [28.0, 35.0]
**Maternal allergy and/or asthma history**			
Positive	5850 (66.3%)	37635 (56.9%)	43485 (58.0%)
Negative	2944 (33.4%)	28272 (42.8%)	31216 (41.6%)
**Maternal smoking status**			
Smoker mother	1633 (18.5%)	9976 (15.1%)	11609 (15.5%)
Non-smoker mother	7122 (80.7%)	55748 (84.3%)	62870 (83.9%)
**Child sex**			
Male	5355 (60.7%)	33015 (49.9%)	38370 (51.2%)
Female	3470 (39.3%)	33110 (50.1%)	36580 (48.8%)
**Pet ownership**			
Pet owner	1541 (17.5%)	9940 (15.0%)	11481 (15.3%)
Non pet owner	7284 (82.5%)	56185 (85.0%)	63469 (84.7%)
**Home age**			
Over 10 years	4393 (49.8%)	31603 (47.8%)	35996 (48.0%)
Not over 10 years	3531 (40.0%)	28484 (43.1%)	32015 (42.7%)
**Main living space cleaning frequency**			
Once a week or more	8138 (92.2%)	60670 (91.8%)	68808 (91.8%)
Less than once a week	680 (7.7%)	5371 (8.1%)	6051 (8.1%)
**Presence of mold in main living space**			
Positive	227 (2.6%)	1340 (2.0%)	1567 (2.1%)
Negative	8598 (97.4%)	64785 (98.0%)	73383 (97.9%)

The chi-square test between asthma incidence and floor materials for all participants provided a P-value of < 0.001. Outcomes of univariable and multivariable regressions for asthma incidence are presented in **Table 2**. The univariable regressions of tatami and carpet over tatami produced odds ratios of 1.09; 95% Confidence Interval (CI) [1.01–1.17] and 1.14; 95% CI [1.06–1.24], respectively. This indicates that the incidence of childhood asthma was 1.09 times higher with tatami flooring and 1.14 times higher with carpet over tatami compared to hard flooring, supporting the conclusion that both tatami flooring and carpet over tatami have positive association with the incidence of childhood asthma. The same trend can be seen in the results of the multivariable regression analysis, which adjusted for potential covariates. The multivariable regression of tatami and carpet over tatami produced odds ratios of 1.09; 95% CI [1.01–1.17] and 1.11; 95% CI [1.02–1.20], respectively.

**Table 2:** Univariable and multivariable regression results.

	Odds ratio	Lower 95% CI*	Upper 95% CI*
Univariable results			
Carpet over hard flooring	0.96	0.91	1.01
Carpet over tatami	1.14	1.06	1.24
Tatami	1.09	1.01	1.17
Multivariable results			
Carpet over hard flooring	0.95	0.90	1.00
Carpet over tatami	1.11	1.02	1.20
Tatami	1.09	1.01	1.17
Univariable results in homes under 10 years old			
Carpet over hard flooring	0.91	0.85	0.98
Carpet over tatami	1.02	0.79	1.30
Tatami	0.97	0.79	1.18
Multivariable results in homes under 10 years old			
Carpet over hard flooring	0.90	0.84	0.97
Carpet over tatami	0.99	0.77	1.27
Tatami	0.96	0.78	1.17
Univariable results in homes over 10 years old			
Carpet over hard flooring	0.97	0.90	1.05
Carpet over tatami	1.09	0.99	1.21
Tatami	1.08	0.98	1.19
Multivariable results in homes over 10 years old			
Carpet over hard flooring	0.97	0.90	1.05
Carpet over tatami	1.09	0.99	1.21
Tatami	1.10	1.00	1.21

The results of the additional regression analysis, stratified by home age of at least ten years or under ten years, are presented in **Table 2.** In homes under ten years old, the chi-square test between asthma incidence and floor materials provided a P-value of 0.036. The univariable and multivariable regressions of tatami produced odds ratios of 0.97; 95% CI [0.79–1.18] and 0.96, 95%CI [0.78–1.17], respectively. Conversely, in homes that were at least ten years old, the chi-square test between asthma incidence and floor materials provided a P-value of 0.080. The univariable and multivariable regressions of tatami produced odds ratios of 1.08; 95% CI [0.98–1.19] and 1.10; 95% CI [1.00–1.21], respectively.

## 4. Discussion

The present study shows that the risk of childhood asthma is increased in homes with tatami mat flooring compared to those with hard flooring, although the risk varies with the age of the building. In addition, no increased asthma risk was observed in carpet over hard flooring compared to hard flooring. These results suggest that geographic differences in indoor environments, particularly flooring types, may influence the incidence of childhood asthma, warranting further research.

The results of the present study are consistent with several previous reports. First, our regression results finding odds ratios of approximately 1.1 for tatami and carpet over tatami are comparable to the odds ratio for tatami on wheezing among Japanese elementary school children reported by Nishima et al. [14]. Our results are similarly consistent with the findings of Yoshino et al., who reported the results of a questionnaire survey conducted among 1410 elementary school students in the 5^th^ grade, finding that children who lived in homes with living room tatami flooring had higher odds ratios for all of the respiratory and allergic disease symptoms measured compared to those who lived in homes with wood flooring [32]. Conversely, they reported odds ratios slightly below 1.0 for "some kind of allergy" and "asthma-like symptoms" among children living in homes with carpet. Furthermore, our findings that tatami flooring increases childhood asthma risk are in agreement with the Japanese allergic rhinitis guidelines, which recommend avoiding tatami mats for allergic rhinitis [33]. However, indoor air allergen levels have also been reported to vary without obvious influence from flooring materials, including carpet, tatami mats, and wood flooring [34]. Accordingly, the reasons for the observed higher incidence of childhood asthma in living spaces with tatami flooring as compared to those with carpet over hard flooring remain unclear.

We therefore took into consideration various perspectives on the potential mechanisms of interaction between tatami flooring and asthma. Multiple studies have investigated candidates for the medium of action between tatami flooring and asthma, one of which is allergic reactions caused by insects, such as house dust mites and booklice [35]. A report from Japan suggested that tatami flooring is correlated with human serum concentrations of IgE specific to booklice, an indoor insect allergen [35]. Furthermore, pollen allergies are associated with asthma incidence [36]. The mesh structure of tatami mats conceivably facilitates pollen accumulation while hindering pollen removal during cleaning. Importantly, pollen exposure is a common source of allergic reactions among Japanese people, as evidenced by a recent nationwide survey which found that over 42% of the population suffers from pollinosis [37].

In contrast to the above, other reports have suggested various benefits of tatami flooring on the indoor environment. The advantages of tatami reportedly include possible antibacterial effects and absorption of harmful substances, such as NO_2_ [38–40]. Moreover, tatami is reported to regulate humidity, and smaller daily variation in humidity was observed in rooms equipped with tatami [41, 42]. Our additional analysis found that the effect of tatami flooring on childhood asthma risk is greater in homes over ten years old, suggesting that older tatami mats correlate with higher risks, while newer mats are not associated with increased risk when compared to hard flooring. It is possible that deterioration in tatami over time may lessen its absorption and moisture-regulating properties, leading to increased dampness, mold growth, and mite proliferation. The nutrients present tatami fibers facilitate mold growth, with mycelia growing within and among the fibers [43]. Collectively, these factors may lead to a greater propensity for mold growth in tatami compared with the other flooring types, which may explain the elevated odds ratios observed for tatami and carpet over tatami.

The present study has several important strengths. Firstly, the JECS is the largest prospective cohort study of its kind conducted in Japan to date, encompassing over 100,000 participants at 15 Regional Centres across Japan [44]. Secondly, JECS researchers from across Japan provided critical review and commentary on this report. Thirdly, our regression analyses were implemented with adjustment for appropriate covariates, according to the DAG [45]. Mite counts and serum IgE levels were deemed intermediate factors associated with house dust, so our analysis was planned without including them as variables in the regression models. Lastly, our results are consistent with previous studies on wheezing, which is the most common asthma symptom [14, 32].

This questionnaire-based study also has several limitations. First, data on asthma history were gathered via self-reporting from respondents, rather than physician-confirmed diagnosis. However, our relevant question item is a validated tool that is frequently used to identify asthma in children, drawn from ISAAC, which was conducted in 56 countries among children aged 6 to 14 years [46]. Next, the questionnaire’s format did not provide detailed information about flooring materials. While traditional tatami is manufactured from Japanese *igusa* straw (*Juncus decipiens*), there is a growing trend toward producing tatami using alternative materials such as paper or resin. However, the questionnaire used in this study did not differentiate among tatami materials. Similarly, we could not distinguish among materials in the hard flooring category, such as hardwood, tile, or vinyl. Of particular importance is the potential presence of polyvinyl chloride (PVC), which is a known childhood asthma risk [47]. Further, it is unclear from this questionnaire whether the reported floor coverings occupy all or only part of the main living space. In addition, the questionnaire did not gather data on the following factors which potentially affect the indoor environment: window size, sunlight exposure, frequency of flipping and replacing tatami mats, and presence of furniture. Lastly, we could not acquire data on dampness, which has recently been reported as a risk factor for asthma. We therefore used the data on indoor mold as a proxy for dampness, although dampness and mold presence are two distinct factors.

## 5. Conclusion

The present study shows that older tatami flooring in the living environment may increase the incidence of childhood asthma. Our results also highlight the importance of evaluating the effects of flooring materials on allergic diseases in a regionally and culturally specific manner.

## Abbreviations/acronyms

CI: Confidence Interval
DAG: Directed acyclic graph
SES: Social economic status
ISAAC: International Study of Asthma and Allergies in Childhood
PVC: Polyvinyl chloride
JECS: Japan Environment and Children’s Study
WHO: World Health Organization
